# Influence of Cycloalkane Carboxylic Acid Ring Size on Tribological Properties of TiN Coatings Under Lubricated Conditions

**DOI:** 10.3390/ma19071394

**Published:** 2026-03-31

**Authors:** Xiaojing Fu, Yaping Fan, Guoxian Jiang, Changsheng Zheng, Yong Wan

**Affiliations:** 1 School of Mechanical and Electrical Engineering, Weifang University of Science and Technology, Weifang 262700, China; 2 School of Mechanical Engineering, Qilu University of Technology (Shandong Academy of Sciences), Jinan 250353, China

**Keywords:** cycloalkane carboxylic acids, tribological properties, carbon-based tribofilm, carbon ring, dissociation, TiN coating

## Abstract

This study aimed to investigate the tribological properties and lubrication mechanisms of three cycloalkane carboxylic acids (cyclopropane, cyclobutane, and cyclopentane) as additives in PAO4 base oil. The testing was conducted in a TiN/steel tribosystem under ball-on-flat reciprocating sliding motion with a maximum contact pressure of 0.9 GPa. Notably, cyclopropane carboxylic acid exhibited excellent anti-friction and anti-wear properties, achieving a microscale coefficient of friction of 0.008 and a wear rate of only 7.0 × 10^−8^ mm^3^/N·m. XPS and Raman analyses of the tribofilms revealed that all three cycloalkane carboxylic acids underwent tribochemical reactions during frictional motion to form carbon-based tribofilms with varying contents. Moreover, cyclopropane carboxylic acid exhibited significant tribochemical reactions, forming carbon-based tribofilm with superior tribological properties. This behavior can be attributed to the stability and size of the carbon ring in cycloalkane, which influences its dissociation and the formation of carbon-based tribofilm under high-temperature and contact stress conditions.

## 1. Introduction

Global industrialization has intensified the demand for energy efficiency and environmental sustainability. Since frictional energy loss and component wear account for approximately 23% of global energy consumption, improving the durability of mechanical systems is crucial for reducing carbon footprints [1,2]. Titanium Nitride (TiN) coatings have been widely adopted across aerospace, automotive, and manufacturing sectors due to their exceptional hardness, high thermal stability, and superior corrosion resistance [3,4,5]. These hard coatings effectively extend the service life of precision components by providing a robust barrier against mechanical degradation [6]. However, TiN coatings are not infallible and often suffer from localized delamination, micro-cracking, and severe abrasive wear when traditional lubricants fail to establish a stable protective interface under extreme boundary lubrication conditions [7,8,9]. Despite their high intrinsic hardness, ceramic coatings require synergistic liquid lubrication to prevent catastrophic surface failure and maintain low friction. Current research indicates that conventional organic friction modifiers often lack the necessary chemical reactivity to provide adequate sacrificial protection for such ceramic-based interfaces [10,11]. Consequently, there is an urgent need to develop novel lubricant additives specifically designed to interact with hard coatings under high-stress conditions [12,13].

Understanding the activation mechanisms of lubricant additives is fundamental to designing high-performance tribosystems [14]. Recent research has shifted the focus from purely thermal activation to mechanochemical processes, highlighting that shear and contact stress play a more decisive role than bulk temperature in initiating tribochemical reactions [15,16]. For instance, Naeini et al. [17] provided atomic-scale evidence through reactive molecular dynamics simulations that molecular dissociation rates significantly increase with normal load and shear stress under confinement. Their findings underscore that mechanochemical activation, rather than bulk temperature alone, governs additive decomposition and subsequent tribofilm formation. This shift in understanding supports the hypothesis that the decomposition of organic molecules at sliding interfaces is a stress-driven process facilitated by mechanical energy input [14,18]. While prior studies have extensively explored various extreme pressure and anti-wear additives [19,20], achieving precise control over the *in situ* reaction kinetics remains a significant challenge. The complexity of the sliding interface necessitates a deeper investigation into how specific molecular architectures can be leveraged to trigger highly efficient tribochemical reactions [21]. This study addresses this critical research question by examining the influence of cyclic structures on the sacrificial protection of TiN coatings.

The intrinsic molecular stability and reactivity of cycloalkanes are fundamentally linked to their internal ring strain [22,23]. This structure–activity relationship is strongly supported by the work of Ma et al. [24], who demonstrated through both experiments and simulations that metastable cycloalkane rings, particularly cyclopropane carboxylic acid (CPCa), undergo more rapid fragmentation and subsequent tribopolymerization compared to more stable analogs. Their findings confirm that high angular strain effectively lowers the energy barrier for ring-opening, facilitating the formation of protective carbon-based tribofilms that shield the substrate from direct contact. Building on this theoretical foundation, we hypothesize that the ring strain of cycloalkane carboxylic acids can serve as a tunable mechanochemical trigger for the synergistic lubrication of hard coatings. In this work, we compare three-, four-, and five-membered ring structures to establish a direct correlation between ring strain and tribochemical reactivity on TiN coatings. This approach aims to provide a novel pathway for designing high-performance lubricants that offer superior sacrificial protection through targeted mechanochemical activation, ultimately moving beyond conventional lubrication frameworks to achieve long-term surface integrity.

## 2. Experimental Section

### 2.1. Materials

**Figure 1:**
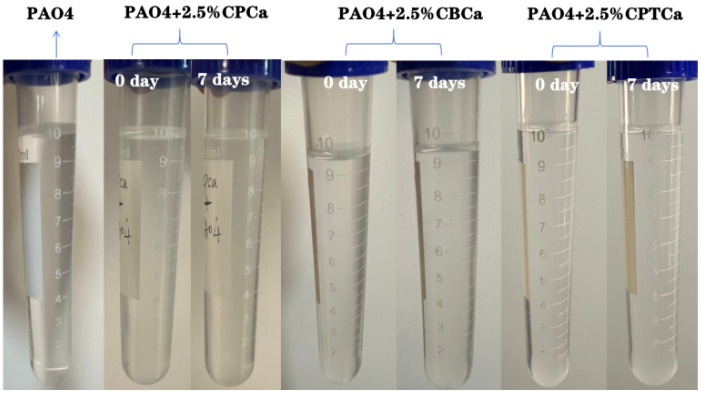
Digital photographs of pure PAO4 base oil and PAO4 containing 2.5 wt.% additive after storage at room temperature.

CPCa, Cyclobutane-carboxylic acid (CBCa), and Cyclopentane-carboxylic acid (CPTCa) with a purity of 98% were commercially purchased as mineral oil additives, respectively. Table 1 lists the molecular structure and basic parameters of these three cycloalkane carboxylic acids. They were separately added to PAO4 base oil (viscosity: 31.7 mPa·s at 20 °C) at a concentration of 2.5 wt.%. To ensure uniform distribution, the mixtures were first subjected to ultrasonic dispersion for 30 min, followed by continuous magnetic stirring for 2 h at room temperature. As illustrated in Figure 1, the lubricant solutions containing various cycloalkane carboxylic acids exhibited excellent compatibility with the PAO4 base oil. Specifically, the PAO4 + 2.5 wt.% additive mixtures remained clear and transparent after 7 d of storage at room temperature. No observable changes, such as discoloration, phase separation, or precipitation, were detected, confirming the robust physical stability of the formulated lubricants. N-hexane (with a purity of 99.5%) was obtained from the Aladdin chemical platform for effective removal of residual oil on both the sample and the dual ball after frictional motion occurred. AISI304 stainless steel flats with an initial roughness of Rq = 28.3 nm were polished to Rq = 20 nm respectively. The dual ball is made of AISI52100 steel with a diameter of 6 mm and a surface roughness of Rq = 28.5 nm.

### 2.2. Characterization of TiN Coatings

As has been shown before, the TiN coatings were deposited onto stainless steel substrates via a standardized magnetron sputtering procedure [25]. The as-deposited TiN coatings had a thickness of approximately 588 nm. The morphological characteristics of both the sample wear and dual-sphere abrasion areas were examined via Scanning Electron Microscopy (SEM, MERLIN Compact). Key mechanical properties of these TiN coatings and the stainless steel and the dual steel ball are summarized in Table 2. Raman spectroscopy (Bruker SENTERRA) was employed to analyze the chemical composition of the tribofilm on the dual-sphere surface, focusing on identifying the carbon-based tribofilm formed on the worn areas. An argon ion laser with an excitation wavelength of 532 nm (2.3 eV) was used, collecting signals between 600 cm^−1^ and 3000 cm^−1^ over 120 s. To avoid surface damage or alteration due to excessive laser energy, the power density was maintained at 0.5 mW/cm^2^. X-ray photoelectron spectroscopy (XPS, PHI-5000 Versaprobe III) analysis was performed to characterize the chemical composition and conduct a semi-quantitative analysis of the tribofilm. The C1s peak at a binding energy of 284.8 eV served as the reference for calibration. The types of compounds and chemical components in the tribofilm were identified via Fourier-transform infrared spectroscopy (FTIR, Bruker Vertex 7). Additionally, the thermal decomposition behavior of the film was evaluated through thermogravimetric analysis (TGA, Netzsch STA 449 F3 Jupiter).

The surface roughness of the TiN coatings was assessed through atomic force microscopy (AFM, Bruker Innova). The cross-sectional profiles of the worn TiN coatings were obtained using a surface profiler (SJ-200, Mitutoyo). Finally, the wear rate was calculated using Equation (1).

(1) $$w_{s}=V/(L\times W)$$

The wear volume (*V*) was calculated through the integration of the cross-sectional areas along the wear track and the multiplication of each area by the length of the stroke. Moreover, the sliding distance (*L*) and the applied load (*W*) were incorporated into the equation.

### 2.3. Tribological Tests

The tribological properties of the TiN/steel tribopair were assessed using various lubricants in a ball-on-plate reciprocating mode with a multifunctional tribometer (CETR UMT-3). The experimental conditions included a normal load of 2 N, corresponding to a maximum contact pressure of 0.9 GPa, a single stroke length of 6 mm, a linear velocity of 120 mm/s, and a standard testing duration of 30 min. These conditions complied with ASTM G99 [26] and ASTM G133 standards [27]. The tribometer provided precise measurements of the coefficient of friction (COF) at sub-millimeter levels within the specified normal load range. The tests were conducted at a temperature of 25 ± 2 °C and relative humidity of 40 ± 5%. Before each test, both the steel ball and specimen were ultrasonically cleaned for 15 min. After cleaning, 30 μL of lubricant was applied to the contact area. All experiments were repeated at least three times to ensure data reliability. The minimum thickness of the lubrication tribofilm was estimated using the Hamrock–Dowson equation:

(2) $$H_{\min}=3.63\frac{U^{0.68}G^{0.49}}{W^{0.073}}(1-e^{-0.68k})$$

where $H_{\min}{=h}_{\min}/R*$, $U=\eta V/E^{\prime }R*$, $G=\alpha E^{\prime }$, and $W=F/E^{\prime }R{*}^{2}$. $h_{\min}$ represent the minimum thickness of the tribofilm. *R* denotes the radius of the steel ball, *η* indicates the bulk viscosity of the lubrication oil containing additives, and *V* represents the average linear velocity during tribological testing. $E^{\prime }$ denotes the effective elastic modulus, *F* signifies the test load, *α* (~18 GPa^−1^ [28]) represents the viscosity–pressure coefficient, and *k* (~1) signifies the ellipticity parameter. Given the low mass fraction (2.5 wt.%) of the cycloalkane carboxylic acid additives and their good compatibility with the base oil, their impact on the pressure–viscosity coefficient of PAO4 is considered negligible. Previous studies on similar organic friction modifiers have shown that such minor additions do not significantly alter the rheological parameters of the base fluid [2,19]. For PAO4 with three cycloalkane carboxylic acids, the theoretical minimum film thickness (*h*_c_) was calculated to be ~11.21–11.58 nm at a 0.9 GPa contact pressure and 120 mm/s sliding speed.

To determine the lubrication regime, the ratio of the theoretical minimum film thickness (*λ*) to the combined surface roughness was evaluated [29].

(3) $$\lambda =\frac{h_{c}}{\sigma }=\frac{h_{c}}{\sqrt{{\sigma_{1}}^{2}+{\sigma_{2}}^{2}}}$$

Here, *σ* denotes the surface roughness of the tribopair, while *σ*_1_ and *σ*_2_ represent the surface roughness of the plate and counter ball after sliding, respectively. The *λ* values obtained for both tribo-systems lubricated with PAO4 and various cycloalkane carboxylic acids were less than 1, indicating that the contact operated in the boundary lubrication regime [30]. According to classical lubrication theory, when *λ* < 1, the lubricant film thickness is insufficient to completely separate the contacting asperities, and direct solid–solid interactions dominate the tribological behavior [31,32]. Under such conditions, friction and wear reduction rely primarily on the formation of chemically or physically adsorbed boundary films rather than on elastohydrodynamic (EHL) pressure-induced fluid films. Furthermore, elastohydrodynamic lubrication typically requires sufficiently high entrainment speed and lubricant viscosity to generate a continuous pressurized fluid film capable of supporting the applied load [33]. In the present study, the relatively low sliding speed and moderate base oil viscosity result in minimal hydrodynamic film build-up. Therefore, the contribution of EHL effects can reasonably be excluded. Table 3 summarizes the physical parameters used for the Hamrock–Dowson film thickness calculation.

## 3. Results and Discussion

### 3.1. Tribological Behavior and Morphological Characterization

**Figure 2:**
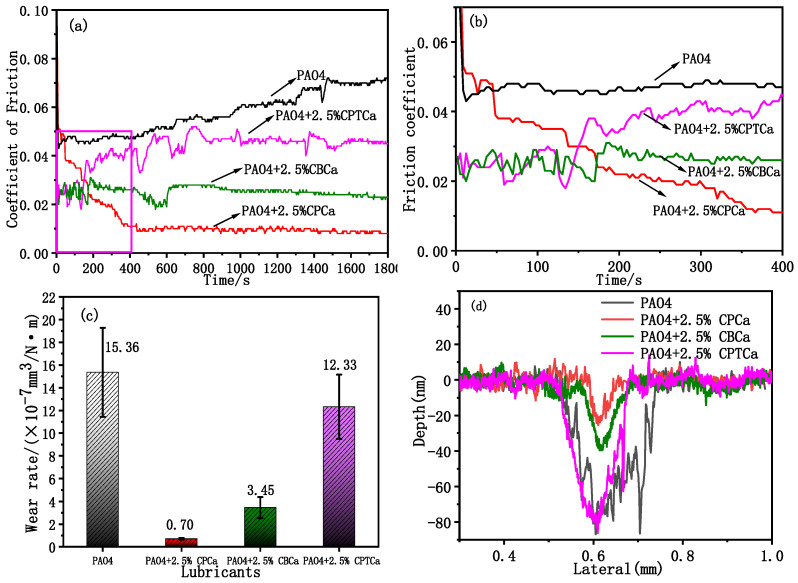
(**a**) COF evolution with sliding time for TiN coatings lubricated with PAO4 base oil and PAO4 containing 2.5 wt.% CPCa/CBCa/CPTCa. (**b**) Partial enlargement of the COF curves in a showing the running-in stage. (**c**) Wear rates of TiN coatings under different lubrication conditions. (**d**) Average cross-sectional topography profile of TiN coating wear tracks under different lubrication conditions (load: 2 N, sliding speed:120 mm/s). The results demonstrate that CPCa exhibits the most significant friction reduction and the lowest wear rate.

Figure 2a,b illustrates the evolution of COF over sliding time for TiN coatings lubricated with PAO4 base oil, both with and without cycloalkane carboxylic acids. During the running-in stage, COF gradually increased and eventually stabilized at 0.072. With the addition of CPCa, CBCa, and CPTCa to the PAO4 base oil, the steady-state COF decreased to 0.008 (88.8% reduction), 0.023 (68.1% reduction), and 0.045 (37.5% reduction), respectively. Therefore, the anti-friction performance of the TiN coating was significantly improved. Among the tested carboxylic acids, CPCa exhibited the highest anti-friction effect, followed by CBCa, which displayed significantly improved anti-friction performance. Under PAO4 + 2.5% CPCa lubrication, COF exhibited a significant exponential decay and rapidly reached a steady-state friction stage after ~400 s, achieving an ultra-low COF of 0.008. Under PAO4 + 2.5% CBCa lubrication, during the running-in stage, COF fluctuated around 0.028 and gradually decreased after ~270 s. Moreover, COF increased gradually and stabilized at 0.023 around 30 s. However, the COF curves for the PAO base oil containing CPTCa were similar to those observed with pure PAO4 base oil. The effect of the three cycloalkane carboxylic acids on the wear performance of TiN coatings exhibited a similar trend consistent with the COF results (Figure 2c). Under PAO4 + 2.5% CPCa lubrication, the TiN coating exhibited a wear rate of 7.0 × 10^−8^ mm^3^/N·m, which was only 4.5% of the wear rate (1.54 × 10^−6^ mm^3^/N·m) observed under pure PAO4 base oil lubrication. In contrast, the wear rates increased to 22.4% and 79.9% under PAO4 + 2.5% CBCa (3.45 × 10^−7^ mm^3^/N·m) and PAO4 + 2.5% CPTCa (1.23 × 10^−6^ mm^3^/N·m) lubrication, respectively, compared with pure PAO4 base oil lubrication. These results indicate that the CPCa additive exhibited significantly superior lubrication performance than the other two additives.

The morphological analysis of the cross-sectional profile of the TiN coating during frictional motion (shown in Figure 2d) revealed that under PAO4 base oil lubrication, the TiN coating exhibited a scratch width of 220 μm and a maximum depth of 88 nm. In contrast, under PAO4 + 2.5% CPCa oil lubrication, the scratch width and the maximum depth decreased to 48 μm and 24 nm, respectively. Under PAO4 + 2.5% CBCa oil lubrication, both the scratch width (129 μm) and maximum depth (38 nm) were smaller than those observed under pure PAO4 oil lubrication. However, under PAO4 + 2.5% CPTCa oil lubrication, the scratch width and maximum depth increased to 213 μm and 83 nm, respectively. Notably, the friction-reduction and anti-wear capabilities of the PAO4 + 2.5% CPTCa lubricant were significantly inferior to those of the CPCa and CBCa systems, with CPCa exhibiting the most outstanding overall performance among all tested additives.

**Figure 3:**
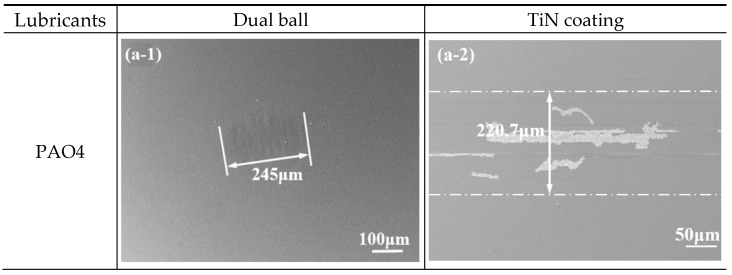

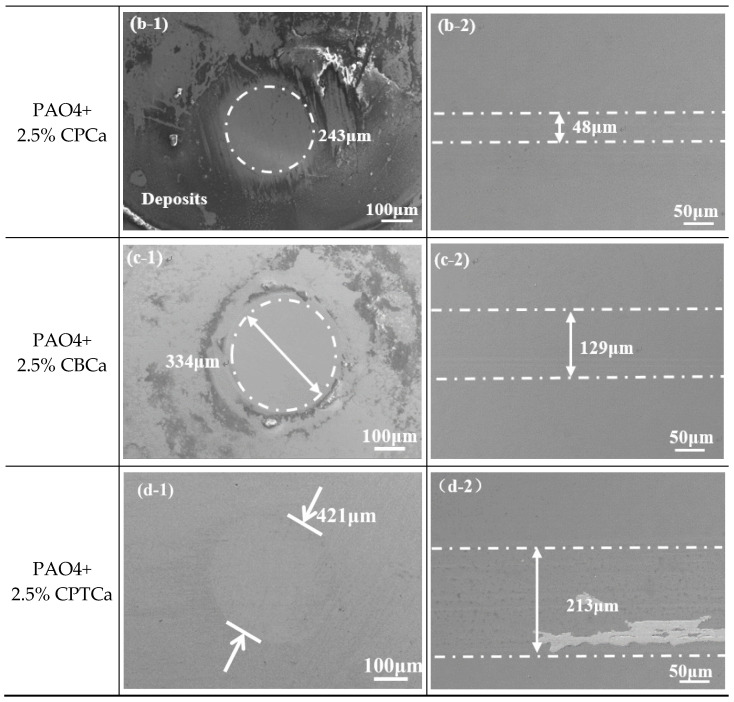
SEM images of wear scars on counterpart steel balls and wear tracks on TiN coatings lubricated with PAO4 and PAO4 + 2.5 wt.% additives. Note: A uniform scale of 100 μm is used for all dual ball images (**a-1**–**d-1**) to encompass the full contact and reaction zones, while a scale of 50 μm is applied to all TiN coating images (**a-2**–**d-2**) to highlight microscopic surface failure modes and track boundaries.

To further evaluate the tribological performance of TiN coatings under different lubrication systems, the wear morphologies of both the coatings and the counterpart steel balls were characterized. Under pure PAO4 lubrication, the wear scar on the steel ball exhibited an elliptical shape with a diameter of 245 μm, characterized by severe abrasive furrows (Figure 3(a-1)). Concurrently, the TiN coating suffered from significant adhesive wear, evidenced by distinct grooves and extensive spalling along the sliding direction (Figure 3(a-2)). However, the addition of cycloalkane carboxylic acids (CPCa, CBCa, and CPTCa) fundamentally altered the wear mechanisms. The wear scars on the counterpart steel balls transitioned to a nearly circular geometry with remarkably smooth surfaces, free from visible mechanical furrows. A notable phenomenon observed across all additive-lubricated samples was that the apparent diameter of the wear scars on the steel balls exceeded the width of the corresponding wear tracks on the TiN coatings. This discrepancy is primarily attributed to the formation of tribochemical reaction products that migrate beyond the physical contact interface [6,34]. Under PAO4 + 2.5% CPCa and PAO4 + 2.5% CBCa lubrication, prominent black deposits were observed around the contact zones, with CPCa exhibiting the most pronounced film formation. In contrast, the wear characteristics under PAO4 + 2.5% CPTCa lubrication represented a transitional state. Although the TiN coating showed localized delamination and scratches (Figure 3(d-2)), the total area of failure was markedly reduced compared to the catastrophic peeling observed with pure PAO4. On the counterpart steel ball (Figure 3(d-1)), the sample lubricated by PAO4 + 2.5% CPTCa exhibited the largest apparent diameter (421 μm). However, this boundary was primarily identified by the distribution of tribochemical reaction products rather than significant material removal, as the physical wear was too minimal to produce discernible grooves. As established by Martin and Ohmae [6], such a feature does not signify exacerbated mechanical wear. Instead, it reflects a diffuse tribochemical interaction zone. Due to the lower ring strain and higher molecular mobility of the five-membered ring structure, the CPTCa-derived species tend to migrate and deposit over a broader area, contrasting with the localized, dense tribofilms formed by CPCa. Consequently, while the protection offered by CPTCa is less localized than that of its three- or four-membered analogs, it still exerts a clear sacrificial lubricating effect that remains significantly superior to the base oil alone.

### 3.2. Characterization of the Tribofilm

**Figure 4:**
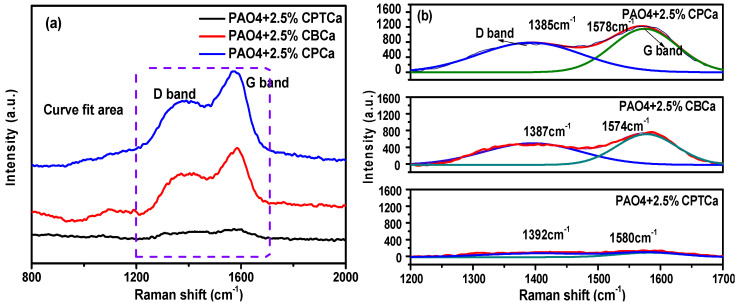
(**a**) Raman spectra of tribofilms formed on the steel ball after tribological testing and (**b**) corresponding Gaussian-fitted Raman spectra of the tribofilms.

To validate the aforementioned speculation, Raman spectroscopy was used to characterize the tribofilms formed on the surface of dual steel balls lubricated with PAO4 oil containing three cycloalkane carboxylic acids. The Raman spectra (Figure 4a) revealed that all three lubricating oils generated tribofilms with carbon-based G and D peaks at ~1580 and 1385 cm^−1^. To further analyze the structure of these tribofilms, Gaussian–Lorentz peak fitting of the Raman spectra was performed (Figure 4b). The PAO4 + 2.5% CPCa, PAO4 + 2.5% CBCa, and PAO4 + 2.5% CPTCa oils exhibited I_D_/I_G_ ratios of 1.47, 1.14, and 1.06, respectively. The Raman analysis of the tribofilms derived from cycloalkane carboxylic acids reveals a significant dependence of the carbon structure on the molecular ring strain. While the positions of the D and G peaks remained relatively constant across the three tribofilms, their I_D_/I_G_ ratios significantly differed, indicating varying structural characteristics in the generated carbon-based films. For the tribofilm formed by PAO4 + 2.5% CPCa oil, the I_D_/I_G_ ratio is higher than that of its analogs. Howerver, a higher I_D_/I_G_ ratio in highly disordered amorphous carbon can sometimes reflect increased structural defects; in the context of the observed G-peak shift and peak narrowing, it signifies an enhanced formation of nanocrystalline graphitic clusters. According to the Ferrari–Robertson model, the transition from Stage 2 (amorphous carbon) to Stage 1 (nanocrystalline graphite) is characterized by an increase in I_D_/I_G_ as the number and size of sp^2^ rings grow [35,36]. It is proposed that the high angular strain in CPCa facilitates the fragmentation and subsequent recombination of carbon atoms into more ordered sp^2^ configurations under shear stress. This more graphitized nature corresponds to the expansion of sp^2^-hybridized domains, which effectively reduces the shear strength of the sliding interface by providing low energy pathways for interlayer sliding. Consequently, the superior lubricity of CPCa is attributed to the formation of a tribofilm with improved structural ordering rather than simple amorphous disorder.

**Figure 5:**
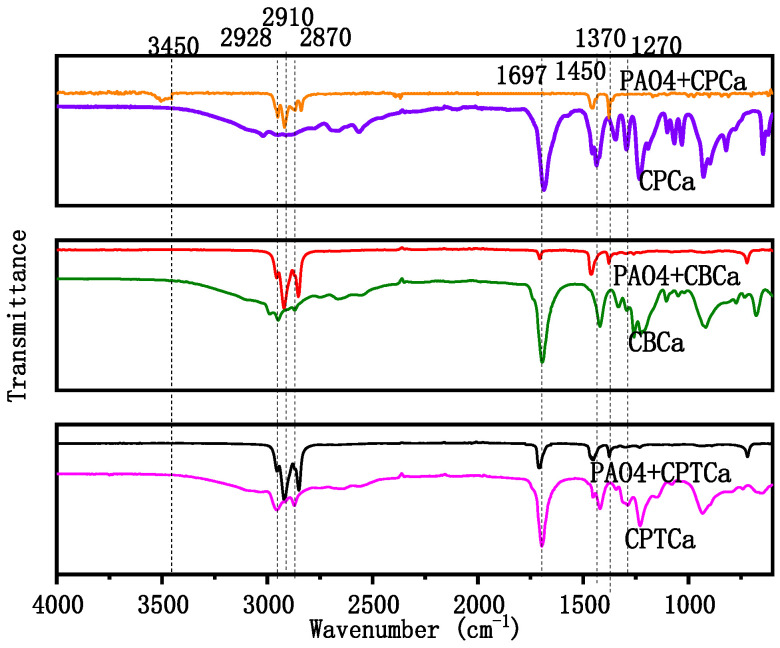
FTIR spectra of tribofilms on the surface of steel ball after tribological testing (including FTIR spectra of pure CPTCa, CBCa and CPCa).

Figure 5 presents the FTIR spectral features of pure CPTCa, CBCa, and CPCa liquids and the tribofilms formed during the dual steel ball friction process with the addition of these liquids to the PAO base oil. Specifically, the FTIR spectra of the tribofilms on the steel ball surfaces and the three cycloalkane carboxylic acid liquids exhibited characteristic absorption peaks at 1270, 1370, and 1450 cm^−1^. These peaks corresponded to C–H bending vibrations. Moreover, characteristic absorption peaks at 2870, 2910, and 2928 cm^−1^ corresponded to C–H stretching vibrations [37]. Notably, the FTIR spectra of all three cycloalkane carboxylic acid liquids exhibited strong C=O stretching vibration peaks. However, in the tribofilms formed by the PAO4 + 2.5% CBCa and PAO4 + 2.5% CPTCa oils, the intensity of this (C=O) peak was significantly reduced and almost undetectable. Additionally, no significant (C=O) characteristic peak was observed in the tribofilms formed by the PAO4 + 2.5% CPCa oil. This experiment provides direct experimental evidence supporting the earlier speculation that the three carboxylic acids undergo divergent chemical pathways during friction.

**Figure 6:**
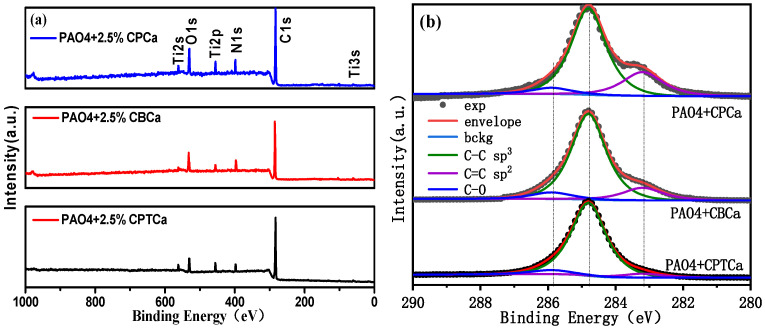
(**a**) Full survey and (**b**) C1s XPS spectra of tribofilms on the surface of TiN coatings.

Figure 6 presents the full survey and C1s XPS spectra of the tribofilms formed on the surface of TiN coating lubricated with PAO4 + 2.5% CPCa, PAO4 + 2.5% CBCa, and PAO4 + 2.5% CPTCa. The tribofilms generated by these three cycloalkane carboxylic acids after frictional motion exhibited similar chemical compositions. Characteristic peaks were observed at binding energies of 283.3, 284.8, and 286.5 eV, corresponding to (C=C) sp^2^, (C–C) sp^3^, and (C–O) functional groups, respectively [38,39]. Additionally, the full width at half maximum values of the C=C (sp^2^) peaks for all three tribofilms were measured at 1.4 eV. Notably, the PAO4 + 2.5% CPCa oil exhibited the highest peak intensity, with a peak area ratio of 22.8%. However, the PAO4 + 2.5% CPTCa oil featured the lowest peak intensity, with a peak area ratio of only 2.7%. These findings suggest that the XPS spectral results were consistent with the FTIR and Raman spectral analyses. This consistency further confirms that the PAO4 + 2.5% CPCa oil exhibited superior lubrication performance at the contact interface owing to its higher graphitic carbon content.

### 3.3. Lubrication Mechanism Analysis

**Figure 7:**
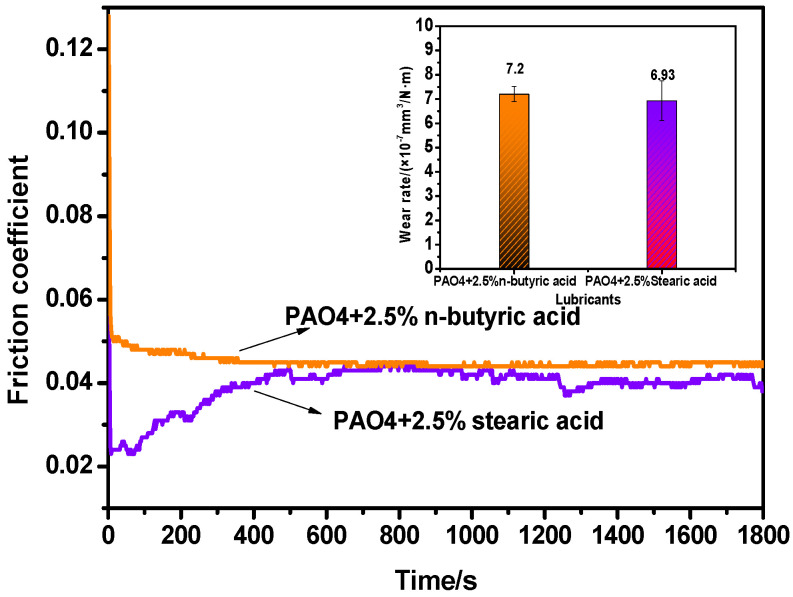
Variation curves of COF with time and the wear rates of TiN coating lubricated with PAO4 + 2.5% n-butyric acid and PAO4 + 2.5% stearic acid (load: 2 N, sliding speed: 120 mm/s).

PAO4 lubricants containing CPCa (a three-membered carbon ring), CBCa (a four-membered carbon ring), and CPTCa (a five-membered carbon ring) exhibited distinct tribological behaviors at identical mass fraction. A comprehensive analysis of Raman spectroscopy, FTIR, and XPS results revealed that these cycloalkane carboxylic acids underwent markedly different degrees of tribochemical reactions during sliding. Notably, CPCa exhibited the highest degree of chemical decomposition and generated the highest amount of carbon-based tribofilm. Although all three additives contained the same surface-active group (−COOH) (Table 1), their distinct behaviors prompted us to investigate whether the carboxyl group alone governed their performance. To decouple the effect of the functional group from that of the cyclic carbon skeleton, short-chain butyric acid and long-chain stearic acid were introduced into PAO4 (Figure 7). The adsorption strength and surface coverage of the carboxylic acids were then analyzed to evaluate whether the −COOH group plays a dominant role in the lubrication performance on TiN. It is well established that carboxylic acids can adsorb strongly on titanium-containing surfaces through coordination between oxygen atoms and surface Ti atoms, forming monodentate or bidentate bonding configurations [40]. Under boundary lubrication conditions, adsorption of polar molecules mainly provides surface anchoring, whereas the long-term friction and wear behavior is primarily governed by stress-activated tribochemical reactions rather than equilibrium adsorption alone [2,41]. Since n-butyric acid and stearic acid possess identical −COOH anchoring groups, their fundamental adsorption mechanism on TiN is expected to be similar. Although differences in alkyl chain length may influence molecular packing density and surface coverage, previous surface chemistry studies have shown that when the anchoring group remains unchanged, the chemisorption strength is predominantly determined by the head group rather than the hydrocarbon chain length [42]. The nearly identical tribological performance observed for these two linear acids therefore suggests that variations in adsorption strength or surface coverage are unlikely to be the dominant factors in the present tribosystem. In contrast, the cyclic carboxylic acids exhibited markedly different tribological behaviors despite sharing the same −COOH functional group. Considering that tribofilm growth under severe contact stress (*λ* < 1) is generally controlled by stress-assisted mechanochemical reactions [32,41], the distinct thermodynamic stability and ring strain of the cyclic backbone are likely to play a key role in regulating stress-induced molecular decomposition kinetics and carbon-based tribofilm formation during sliding.

Previous studies have reported the average combustion heat per (CH_2_) unit and the corresponding angular strain energies for ethylene and several cycloalkanes [43]. Cyclopropane and cyclobutane exhibited higher angular strain energy than ethylene. Under high-temperature or catalytic conditions, these two compounds were more prone to conversion into more stable alkenes [43]. Ring size fundamentally governs cycloalkane stability through modulation of ring strain [44]. According to chemical bonding theory [43,45], covalent bond strength depends on effective orbital overlap and optimal bond angles. For sp^3^-hybridized carbon atoms, the ideal bond angle is 109.5°, corresponding to a tetrahedral configuration. Deviation from this ideal geometry reduces orbital overlap efficiency, weakens C-C bond strength, and increases internal strain energy. In cyclopropane, the carbon atoms form an equilateral triangular ring with C-C-C bond angles constrained to approximately 60°. This severe deviation from the tetrahedral angle prevents effective head-on orbital overlap. Consequently, the bonds adopt a bent configuration [46], resulting in weakened C-C bonds, high ring strain, and enhanced susceptibility to ring-opening reactions. If cyclobutane were perfectly planar, its C-C-C bond angle would be 90°, still smaller than the ideal value. Although angular strain is present, it is less severe than in cyclopropane. In reality, cyclobutane adopts a nonplanar folded conformation, increasing the bond angle to approximately 111.5° and thereby reducing strain. As a result, cyclobutane is more stable than cyclopropane. Cyclopentane has an internal bond angle of 108° in a planar pentagonal configuration, which is close to the ideal tetrahedral angle. To further minimize strain, cyclopentane adopts a non-planar envelope conformation, reducing bond bending and enhancing stability. These intrinsic structural differences provide the thermodynamic basis for the distinct mechanochemical reactivity observed during sliding.

**Figure 8:**
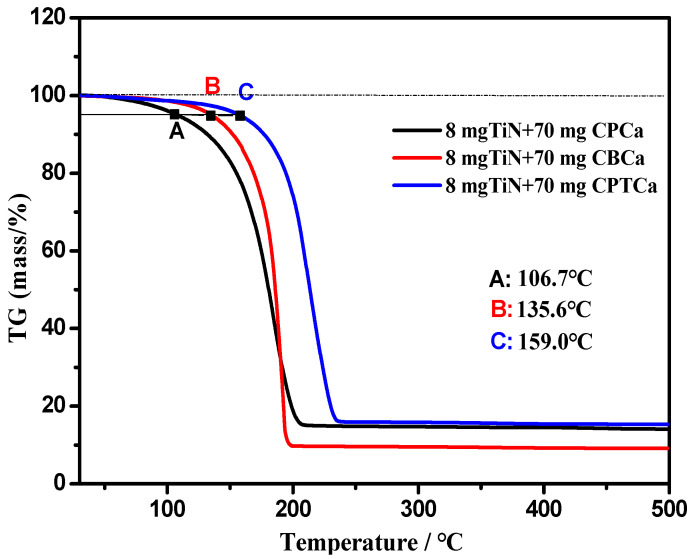
Thermal degradation behavior of cycloalkane carboxylic acid additives mixed with TiN powder. The TGA curves reveal a distinct correlation between molecular ring strain and thermal stability, with CPCa exhibiting the earliest mass loss, indicating its relatively lower thermodynamic stability compared to CBCa and CPTCa.

TGA was used to assess the thermal stability of the three cycloalkane carboxylic acids. This TGA (see Figure 8) presents a comparative evaluation of the thermal degradation behavior of three distinct sample compositions: namely, a mixture of 8 mg TiN powder with 70 mg CPCa, 8 mg TiN powder with 70 mg CBCa, and 8 mg TiN powder with 70 mg CPTCa. The plot’s abscissa indicates temperature (in °C), while the ordinate denotes the percentage of mass retention. The relative thermal stability of each sample was determined by identifying the characteristic temperatures at which substantial mass loss commenced under a controlled heating process [47]. As evidenced by the TGA curves, all samples underwent progressive mass loss with increasing temperature; however, a notable distinction was observed in their respective initial decomposition temperatures. Specifically, the formulation incorporating CPCa exhibited a 5% mass loss at a considerably lower temperature of 106.7 °C, revealing its relatively inferior thermal stability [48]. Conversely, the blends containing CBCa and CPTCa demonstrated enhanced resistance to thermal degradation, achieving the same 5% mass loss threshold at higher temperatures of 135.6 °C and 159.0 °C, respectively. This divergence clearly indicates that CPCa possesses the least thermal stability within this series of cyclocarboxylic acids, likely attributable to the decomposition of its molecular structure initiating at lower thermal energy.

To further distinguish the driving forces behind tribofilm formation, it is essential to decouple thermal and mechanochemical effects. Given the low bulk temperature (25 °C) and the calculated film thickness ratio (*λ* ≈ 0.35), the tribosystem operates strictly within the boundary lubrication regime where frictional energy is concentrated at asperity contacts. Under these conditions, the intrinsic thermal stability of the additives is unlikely to be the primary limiting factor, since the bulk temperature remains far below their decomposition temperature, suggesting that thermally driven activation alone is insufficient to dominate the reaction pathway. According to Naeini et al. [17], the activation of such reactive species is primarily driven by the mechanical work performed by shear stress and normal load under confinement. The high ring strain of CPCa effectively lowers the mechanochemical activation barrier for mechanochemical dissociation compared to the more stable four- or five-membered analogs [24]. This allows CPCa to fragment more readily through ring-opening reactions triggered by mechanical energy input rather than thermal fluctuations. Furthermore, the TiN surface plays a dual role as both a mechanical support and a reactive template [6]. It provides specific binding sites that facilitate the catalytic conversion of these high-energy fragments into a protective, cross-linked carbon-based network. This synergistic process, referred to as stress-induced surface catalysis [49], demonstrates that the tribochemical performance of CPCa is a combined result of its high intrinsic ring strain, the intense shear environment, and the catalytic TiN interface.

In this study, three cycloalkane carboxylic acids soluble in PAO4 base oil were selected. CPCa and CBCa containing three- and four-membered carbon rings are metastable and prone to decomposition at low temperatures and pressures [50]. In contrast, the five-membered carbon ring in cyclopentane carboxylic acid is highly stable. Therefore, during sliding, CPCa and CBCa can effectively reduce friction through the *in situ* formation of carbon-based tribofilm, indicating excellent lubrication performance on TiN coatings. Among the three acids, CPCa exhibited the highest overall lubrication performance. It should be noted that the above tribochemical mechanism and the superior lubrication performance of CPCa are derived from the typical boundary lubrication conditions designed in this study, and the tribological behavior of these cycloalkane carboxylic acid additives may vary with the changes in contact pressure, sliding speed and additive concentration.

## 4. Conclusions

This study aimed to investigate the structure–performance relationship of cycloalkane carboxylic acids (CPCa, CBCa, and CPTCa) as lubricant additives for TiN coatings and to elucidate the underlying tribochemical mechanisms governing *in situ* carbon-based tribofilm formation. The main findings are summarized as follows:

1. CPCa demonstrated exceptional tribological performance, achieving an ultra-low coefficient of friction (0.008) and a specific wear rate of 7.0 × 10^−8^ mm^3^/N·m under a contact pressure of 0.9 GPa, outperforming its larger-ring analogs.
2. Surface characterization (XPS, Raman and FTIR) confirmed that all additives participate in tribochemical reactions to form carbon-based tribofilm. The extent of graphitization and the resultant film quality are positively correlated with the tribological enhancement.
3. A distinct structure–activity relationship was established: the anti-friction and anti-wear efficacy decreases with increasing carbon ring size (C3 > C4 > C5), directly linking molecular ring strain to tribochemical reactivity.
4. The superior performance of CPCa is attributed to the high angular strain of its three-membered ring, which significantly lowers the mechanochemical activation barrier for stress-induced ring-opening and facilitates the formation of a highly graphitized, protective tribofilm under severe contact conditions.

This work establishes a significant structure–activity relationship by demonstrating that molecular ring strain can serve as a tunable energy trigger to control the initiation and kinetics of *in situ* tribochemical reactions. This concept offers substantial potential for the rational design of high-performance phosphorus- and sulfur-free additives for advanced ceramic and metallic coatings in modern automotive and industrial systems.

## Figures and Tables

**Table 1:** Molecular structure and basic parameters of three cycloalkane carboxylic acids.

Additives	Chemical Formula	Purity (%)	Flash Temperature (°C)	Melting Point (°C)
CPCa	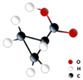	98	71	14~17
CBCa	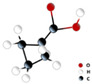	98	83	−7.5
CPTCa	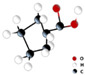	98	93	4

**Table 2:** Mechanical properties of the TiN coating, the stainless steel and the dual steel ball.

	Nano-Hardness (GPa)	Elastic Modulus (GPa)	Roughness (nm)
AISI304 stainless steel	6	193	20.0
TiN coating	26	329	1.5
Steel ball	9	210	28.5

**Table 3:** Physical parameters and calculated results for PAO4 lubricant.

Parameters	Symbol (Unit)	Value
Viscosity–pressure coefficient	*α* (GPa^−1^)	18.0
Viscosity (at 20 °C)	*η* (mPa·s)	31.7
Sliding speed	*u* (m/s)	0.12
Normal load	*F* (N)	2.0
Effective elastic modulus	$E^{\prime }$ (GPa)	234.8
Calculated center film thickness	*h*_c_ (nm)	10.98
Composite surface roughness	*σ* (nm)	~32.4
Film thickness ratio	*λ*	0.34

## Data Availability

The original contributions presented in this study are included in the article. Further inquiries can be directed to the corresponding authors.
